# Structural and optical properties of SnO nano-filler in eco-friendly PVA polymer for flexible optoelectronic applications

**DOI:** 10.1038/s41598-025-14376-3

**Published:** 2025-08-20

**Authors:** Hoda El-Nagar, M. S. Abd El-Sadek, E. M. M. Ibrahim, Sahar Elnobi

**Affiliations:** 1 Nanomaterials Lab., Physics Department, Faculty of Science, South Valley University, Qena, 83523 Egypt; 2 Physics Department, Faculty of Science, Galala University, Galala City, Egypt; 3 Physics department, faculty of science, Sohag University, Sohag, 82524 Egypt; 4 Physics Department, Faculty of Science, South Valley University, Qena, 83523 Egypt

**Keywords:** SnO, Polymer, Optical properties, Flexible optoelectronic application, Materials science, Optics and photonics, Physics

## Abstract

Polyvinyl Alcohol (PVA) has garnered significant attention in the field of flexible optoelectronics due to its unique properties. This study investigates the effect of incorporating tin oxide (SnO) nanoparticles (NPs) with various concentrations (0, 2, 3, 4, and 5 wt%) on structural, optical, and dielectric properties of PVA films synthesized via the solution casting technique. XRD analysis revealed a 28% increase in crystallite size (from 25.74 to 32.88 nm) and reduced dislocation with rising SnO content, indicating enhanced structural ordering. Scanning electron microscopy (SEM) was confirmed homogeneous nanoparticle (NP) distribution at ≤ 3 wt% but aggregation for 5 wt%. Fourier-transform infrared (FT-IR) and Raman spectroscopy were verified hydrogen bonding between SnO and PVA hydroxyl groups. Optical band gap energy was decreased systematically from 4.59 eV (pure PVA) to 4.18 eV (5 wt% SnO), confirming enhanced semiconducting behavior. Photoluminescence (PL) intensity was quenched significantly own to SnO-PVA cross-linking, with new SnO-related emission peaks emerging at 432 nm. The dispersion and the dielectric parameters were determined as functions of SnO concentrations. Nonlinear optical susceptibility (χ ^(3)^) and nonlinear refractive index (n_2_) rushed to 48.84 × 10^−15^ and 11.43 × 10^−13^ esu, respectively for 5 wt% SnO film, demonstrating strong potential for nonlinear devices. These results highlight PVA/SnO films as promising candidates for flexible optoelectronics application.

## Introduction

 Polymers are crucial to advancing optical communication technologies due to their versatility, cost-effectiveness, and performance benefits across a wide range of photonic applications^1^. Over the past decade, there has been a significant increase in the utilization of dye-doped polymers due to their numerous advantages, particularly in linear and nonlinear photonic devices^2,3^. Recently, researchers have increasingly focused on evaluating polymers’ optical and electrical properties. Their enhanced polarization, reflection, anti-reflection, and interference properties are crucial to numerous technologies, such as optical devices^4–6^. Among many fundamental and widely utilized polymers, polyvinyl alcohol (PVA) stands out for its low flammability and extraordinary chemical resistance^7^. PVA is mostly utilized as a finishing and sizing agent in the textile industry^8^. PVA, a synthetic linear polymer, displays noteworthy water solubility, high optical transparency, and excellent film-forming capabilities. Its inherent biocompatibility, biodegradability, and non-toxicity further improve its utility. These combined properties processability from aqueous solutions, optical clarity, non-corrosiveness, robust film formation, biocompatibility, and biodegradability enable diverse applications. Particularly, PVA is incorporated into water-soluble fabrics for producing biodegradable items such as protective clothing, wipes, sponges, sheets, hospital laundry bags, covers, and other physiological hygiene products^9–11^. The optical and electrical features of polymers can be changed by adding dopants like nanoparticles, organic dyes, quantum dots, and other substances that work with the main material. Consequently, polymer-nanoparticle (PNP) composites have garnered significant research interest as a versatile platform for tailoring material properties to meet specific application requirements. These composite systems exhibit enhanced multifunctional properties including thermal, mechanical, optical, and electrical characteristics compared to pure polymers. Moreover, the incorporation of nanoparticles can improve the amorphous nature of the synthesized PNP, contributing to these performance improvements^12–17^. The efficacy of nanocomposite materials in optoelectronic applications can be assessed through a comprehensive investigation of optical characteristics like optical energy gap, optical constants, and dielectric constants, which can be derived from the spectroscopic characteristics of PVA nanocomposite films. These parameters are crucial for several different uses^18–21^.

Tin oxides can be utilized as dopants/fillers to modify structural, physical properties of PVA. Research has demonstrated that the incorporation of tin oxides into PVA matrices leads to significant alterations in its properties, enhancing its suitability for a wide variety of applications, including optoelectronics, sensors, and functional coatings^22–25^. Among tin oxides, SnO received considerable attention for photonic applications due to its p-type conductivity, and high theoretical specific capacity^26,27^. In contrast to SnO₂, SnO exhibits distinct physicochemical properties and applications. Notably, SnO is cost-effective, naturally abundant, and possesses high optical transparency combined with low electrical resistivity. These characteristics render SnO a promising candidate for next-generation transparent flexible electronics devices. Additionally, SnO is well-suited for use in electrochromic devices, solar cells, and transparent conductive oxide (TCO)^28–32^. Its high sensitivity to low gas concentrations further makes it an effective material for gas sensing applications^32^.

To the best of our knowledge, there have been no studies to date that describe the targeted modification of structural and linear/nonlinear optical properties in PVA matrices using SnO NPs, which remains underexplored. This research gap is significant, as SnO NPs offer distinct advantages over the more commonly studied tin dioxide (SnO₂), notably their inherent p-type conductivity, a critical requirement for emerging flexible optoelectronic applications such as transparent transistors, hole-transport layers in photovoltaics, and p-type elements in complementary circuits. Thus, successfully blending SnO NPs into PVA to harness these properties while maintaining even distribution, consistent film quality, and easy processing presents a major challenge. These include mitigating nanoparticle aggregation and controlling interfacial interactions. This work systematically addresses this gap by investigating the fundamental influence of SnO NPs concentration on the structure-property relationships within PVA nanocomposites, aiming to unlock their potential for advanced optoelectronic devices.

## Experimental techniques

### Preparation of PVA film

 PVA ([CH_2_CH (OH)] n) was sourced from LOBA Chemie, and tin oxide (SnO) was acquired from Sigma Aldrich. PVA and PVA–SnO nanocomposite films were fabricated using the solution casting method. Initially, 2 g of PVA were dissolved in 50 ml of deionized water. The mixture was stirred at 75 °C with a stirring speed of 900 rpm for 3 h. To prepare PVA-SnO nanocomposite films, SnO nanoparticles were incorporated into the PVA solution at concentrations of 0, 2, 3, 4, and 5 wt% relative to PVA weight. To promote dispersion and mitigate nanoparticle agglomeration, each mixture was sonicated and subsequently stirred at 30 °C and 900 rpm for 1 h prior to casting. The resulting homogeneous solutions were cast onto Petri dishes and dried in a convection oven at 55 °C for 24 h (see Schematic 1).

**Schematic 1:**
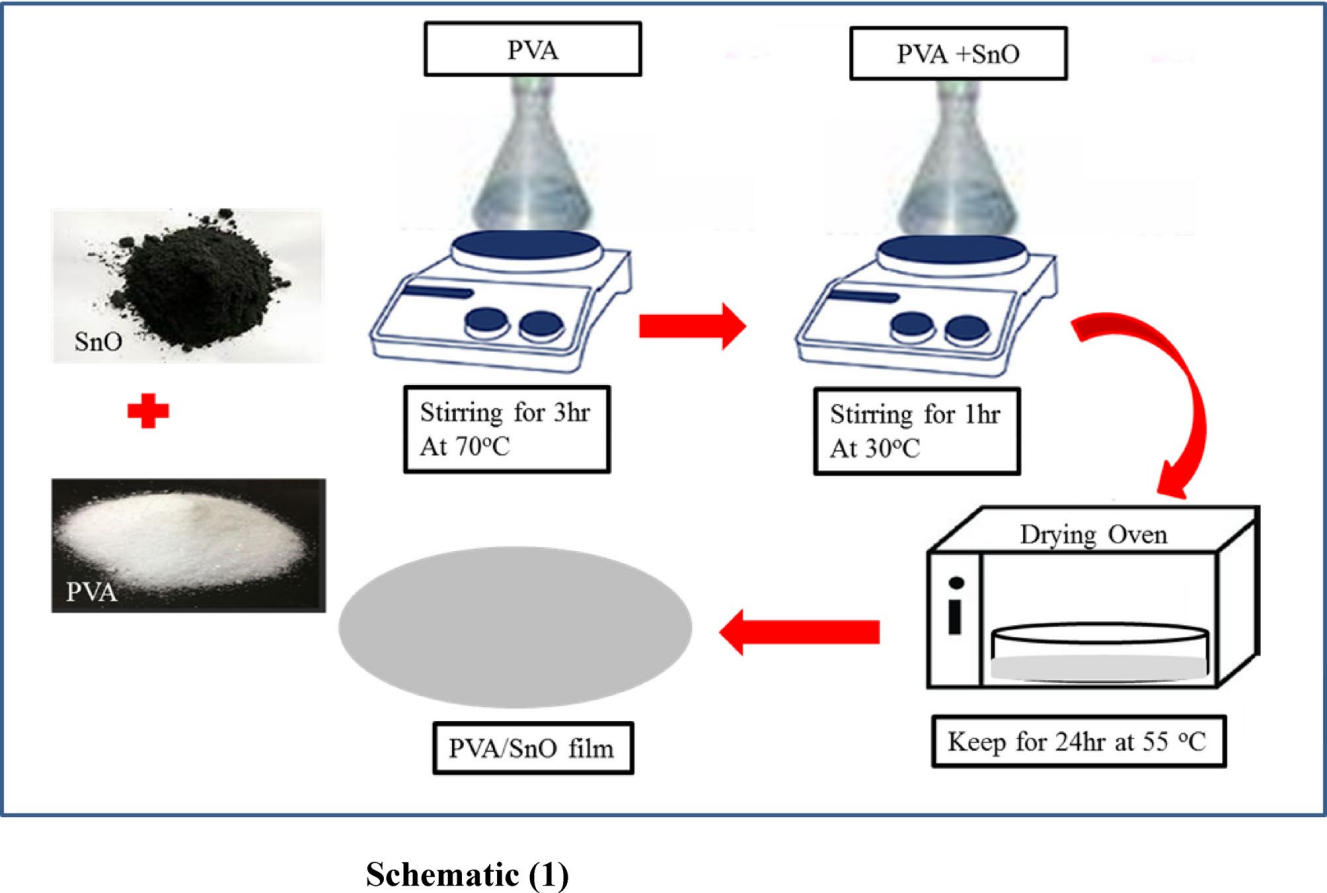
A schematic diagram of the solution casting Method.

The weight% (wt %) of SnO nanoparticles in the PVA matrix was determined using the following formula^33^:

1 $$\:{\text{w}}_{SnO}{\%}=\frac{{\text{w}}_{SnO}}{{\text{w}}_{P}+{\text{w}}_{SnO}}\text{x}\:100$$

where, $\:{\text{w}}_{SnO}$​ represents the weight of the SnO nanoparticles and $\:{\text{w}}_{P}$​ denotes the weight of the PVA polymeric blend. This approach ensures a uniform distribution of SnO nanoparticles within the PVA matrix, facilitating the investigation of their influence on the composite’s properties.

### Material characterization

The crystal structures of all materials were analyzed using XRD technique (XRD, Bruker D8 Advance with a monochromatic Cu-K_α_ radiation source λ = 1.54056 Å) operating at 40 kv and 60 mA in 2θ = 20–70^o^ range. In order to examine the functional groups, FTIR spectra were recorded using a Bruker VERTEX 80 combined Platinum Diamond ATR, which consists of a diamond disc as an internal reflector in the range of 4000–400 cm^−1^ with resolution 4 cm^−1^. The Raman spectrum of the PVA/SnO films was examined by Raman spectrometer (Jasco NRS-2100) at room temperature in the spectral range between 80 and 3200 cm^−1^. Using a SEM model Quanta FEG250 and an EDS, morphology investigation and elemental quantification were carried out, respectively. The optical properties of samples were analyzed by UV–Vis spectroscopy using a (JASCO 670 UV–vis-NIR) spectrophotometer within wavelength range of 200–2500 nm. The emission spectra of the same sample were also measured using a JASCO FP-8500 spectrofluorometer, with an excitation wavelength set at 330 nm.

## Results and discussion

### Structure properties of PVA/SnO films

**Fig. 1:**
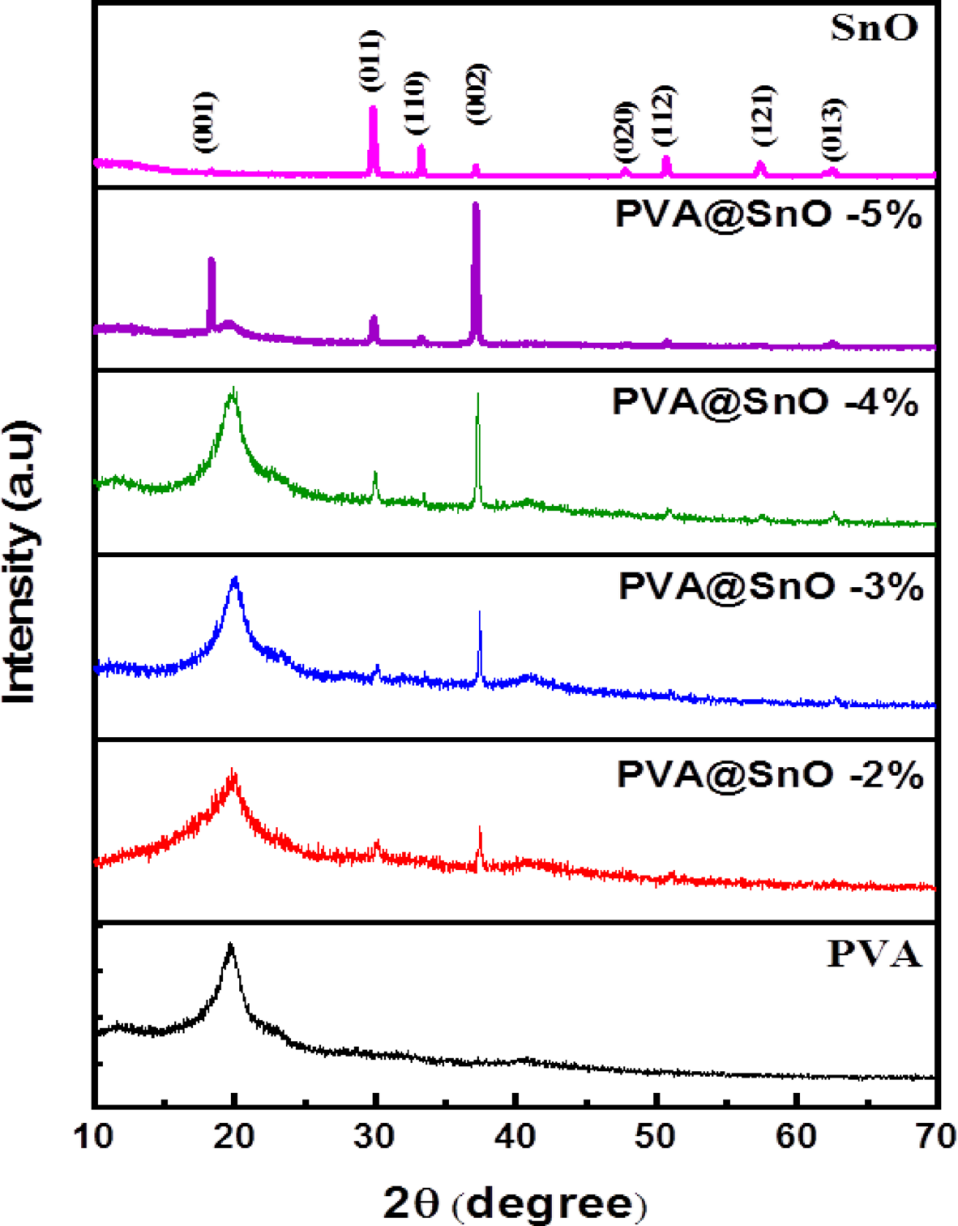
XRD spectra of PVA/SnO nanocomposites films with different ratios of SnO.

Figure 1 shows the XRD for pure PVA film, SnO powder and PVA doped with different concentrations of SnO (2, 3, 4 and 5%). The data imply that for pure PVA a broad peak centered at 2θ = 19.64º is observed which indicates the semi- crystalline nature of the polymer film^34^. Strong intermolecular hydrogen bonding inside each monomer unit of PVA and intermolecular hydrogen bonding between its various monomer units might be the cause of this^35^. The SnO sample exhibits pure tetragonal structure with orientation at 18.3, 29.87, 33.3, 37.1, 47.8, 50.7, 57.4, and 62.48^o^ that fit according to pattern: COD 9,012,140 with orientation in (001), (011), (110), (002), (020), (112), (121), and (013) planes, respectively^36,37^. The PVA peak (at 2θ = 19.64º) is diminished by increasing the content of SnO from 2 to 5%. Also, the intensity of the SnO peaks increases by increasing the SnO content in films. Scherrer’s equation is demonstrated to get the average crystallite size, D as following^38^:

**Fig. 2:**
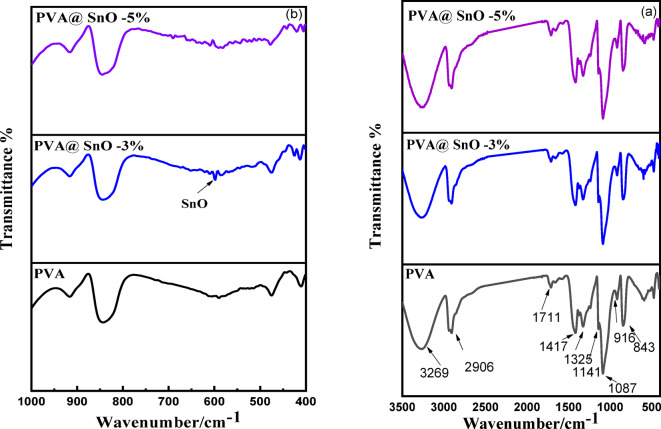
FTIR spectra for pure PVA and PVA/SnO nanocomposites films in the wavenumber range: 400–3500 cm^−1^ **(a)** and 400–1000 cm^−1^ **(b)**.

2 $$\:\text{D}=\frac{\text{K}{\uplambda\:}}{{\upbeta\:}\text{cos}{\uptheta\:}}$$

where, λ is the CuKα X-ray wavelength (0.15406 nm), θ is the Bragg’s angle, β is the Full Width at Half Maximum (FWMH) in radians, and K = 0.94 is Scherrer’s constant. The FWHM of the diffraction peak for the (011) plane clearly shows the nanosize nature of the nanocomposite film. The following formulas are used to calculate the other microstructural parameters for PVA/SnO nanocomposite films. The dislocation density, δ, which is the length of dislocation lines per unit volume, is given in the next relationship^38^:

3 $$\:{\updelta\:}=\raisebox{1ex}{$1$}\!\left/\:\!\raisebox{-1ex}{${D}^{2}$}\right.$$

The following relation is used to compute the micro strain, $\:{{\upepsilon\:}}_{s}$^39^:

4 $$\:\raisebox{1ex}{${{\upepsilon\:}}_{s}={\upbeta\:}\text{cos}\theta\:$}\!\left/\:\!\raisebox{-1ex}{$4$}\right.$$

Nc, the amount of crystallites per unit surface area, is calculated using the following relation^40^:

5 $$\:{\text{N}}_{c}=\raisebox{1ex}{$d$}\!\left/\:\!\raisebox{-1ex}{${D}^{3}$}\right.$$

In this context, d (~ 200 ± 5 μm) refers to the thickness of the sample. Table 1 lists values of D, δ, ε_s_, and N_c_ for various concentrations of SnO. It is evident that when the concentration of SnO increases, the crystallite size values are raised from 25.74 to 32.88 nm, while δ, ε_s_, and N_c_ values are decreased. As the concentration of SnO increases, more point defects are eliminated from the films, and the growth of crystallites along with fewer grain boundaries shows that high-quality PVA/SnO nanocomposite films are being made^41^.

**Table 1:** Structural parameters for pva/sno film.

Films	D (nm)	δ (lin m)^−2^ × 10^15^	ε × 10^−3^	*N*_c_ (m^−2^)×10^18^
PVA − 2%SnO	25.74	1.51	1.406388	5.86
PVA − 3% SnO	28.43	1.24	1.273362	4.35
PVA − 4%SnO	30.17	1.09	1.199825	3.64
PVA − 5% SnO	32.88	0.92	1.101002	2.81

The FTIR spectra of PVA and PVA/SnO nanocomposite films recorded within the wavenumber range of 400–3500 cm^−1^ are shown in Fig. (2a). To effectively distinguish between different peaks and clearly show the SnO peak, this spectrum is displayed in two figures: 400–3500 cm^−1^ in Fig. (2a) and 400–1000 cm^−1^ in Fig. (2b). In Fig. (2a) the typical transmittance peaks for PVA are located at 3269 cm^−1^ (O–H stretching), 2906 cm^−1^ (asymmetric stretching of CH_2_), 1711 cm^−1^ (water absorption), 1417 cm^−1^ (bending of CH_2_), and 1325 cm^−1^ (δ(OH), rocking with CH wagging)^34^extending the C-O shoulder stretching in the crystalline sequence of PVA at 1141 cm^−1^, whereas the amorphous sequence of PVA involves bending the OH and stretching of C–O at 1087 cm^−1^, 916 cm^−1^ (CH_2_ rocking), 843 cm^−1^ (C–C stretching)^42^. It was expected that the nanostructured Sn–O sharp peak would exhibit a stretching vibration at 598 cm^−122^ as show in Fig. (2b).

**Fig. 3:**
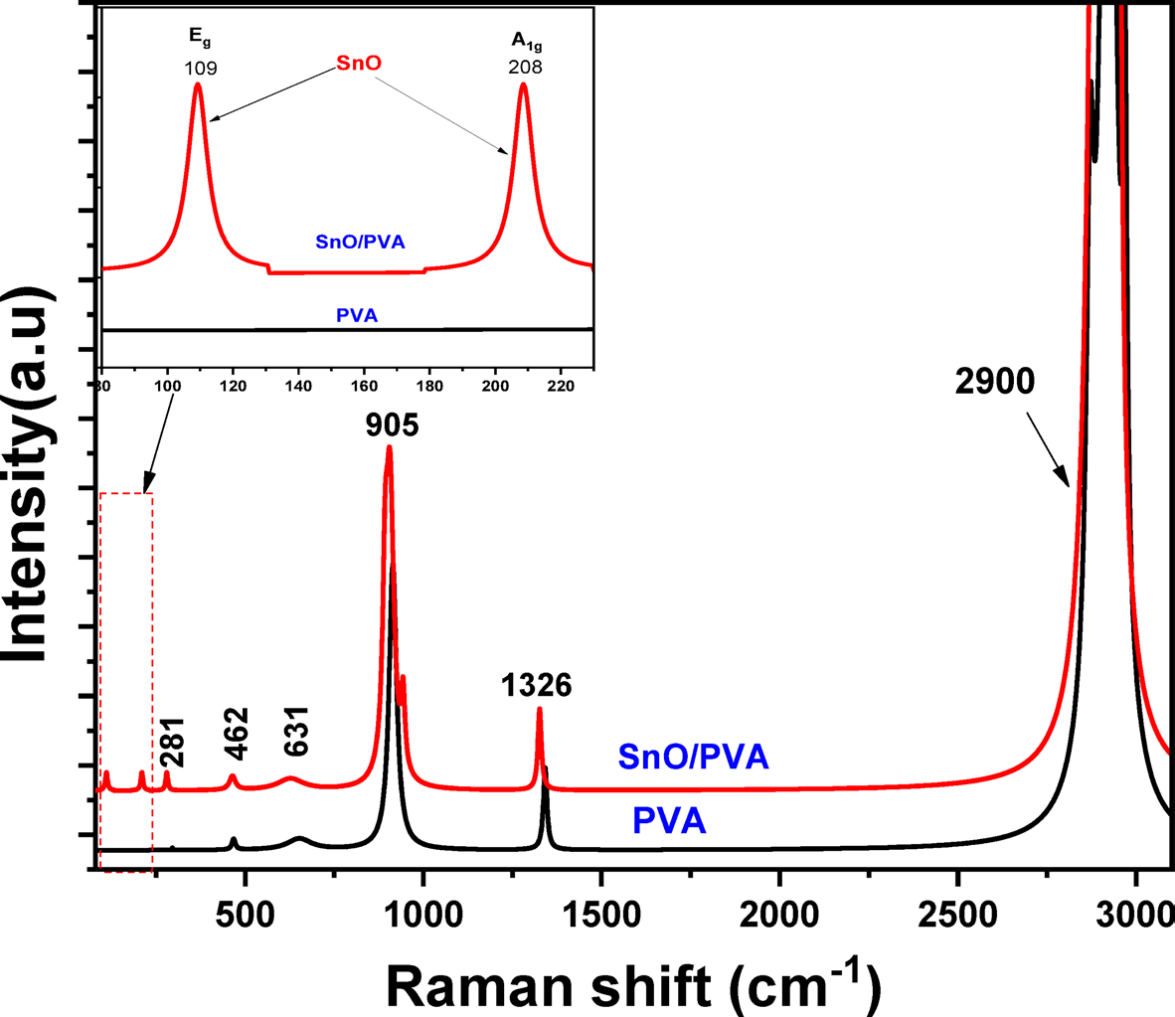
Raman spectra for pure PVA and PVA/SnO (5%) film.

Raman spectroscopy provides a dependable method for detecting the presence of nanoparticles and examining their interactions with polymers by analysing variations in peak locations and intensities. The spectra of SnO (5%)-doped PVA and pure PVA films (see Fig. 3) exhibit characteristic vibrational modes, including a prominent band near 2900 ± 10 cm⁻¹ attributed to the C–H stretching vibrations of –CH₂ groups in the PVA matrix^43–45^ as well as additional vibrational features such as 1325 ± 10 cm⁻¹ (CH₂ wagging/C–H bending), 903 ± 5 cm⁻¹ (C–C stretching), 636 ± 5 cm⁻¹ (C–O–C bending), 466 ± 3 cm⁻¹ (C–C bending), and 280 ± 3 cm⁻¹ (skeletal bending), appeared in the SnO-doped PVA and undoped sample. When SnO is added, this mode shifts in position and strength, showing that the SnO nanoparticles are interacting with the PVA matrix and changing its local chemical environment. Notably, the emergence of SnO-specific E_g_ and A_₁g_ modes at 109 and 208 cm⁻¹^26,27^respectively, upshifted relative to bulk SnO, confirms successful incorporation.

**Fig. 4:**
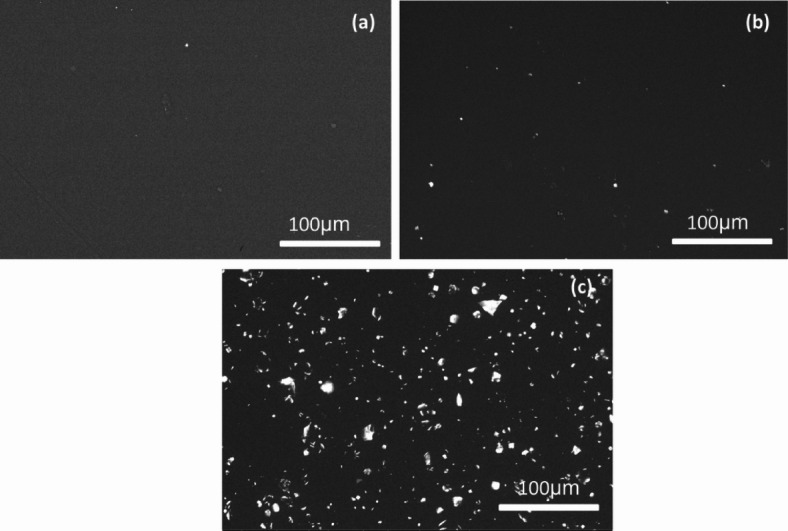
SEM images for PVA **(a)**, PVA/SnO − 3% **(b)** and PVA/SnO − 5% **(c)**.

**Fig. 5:**
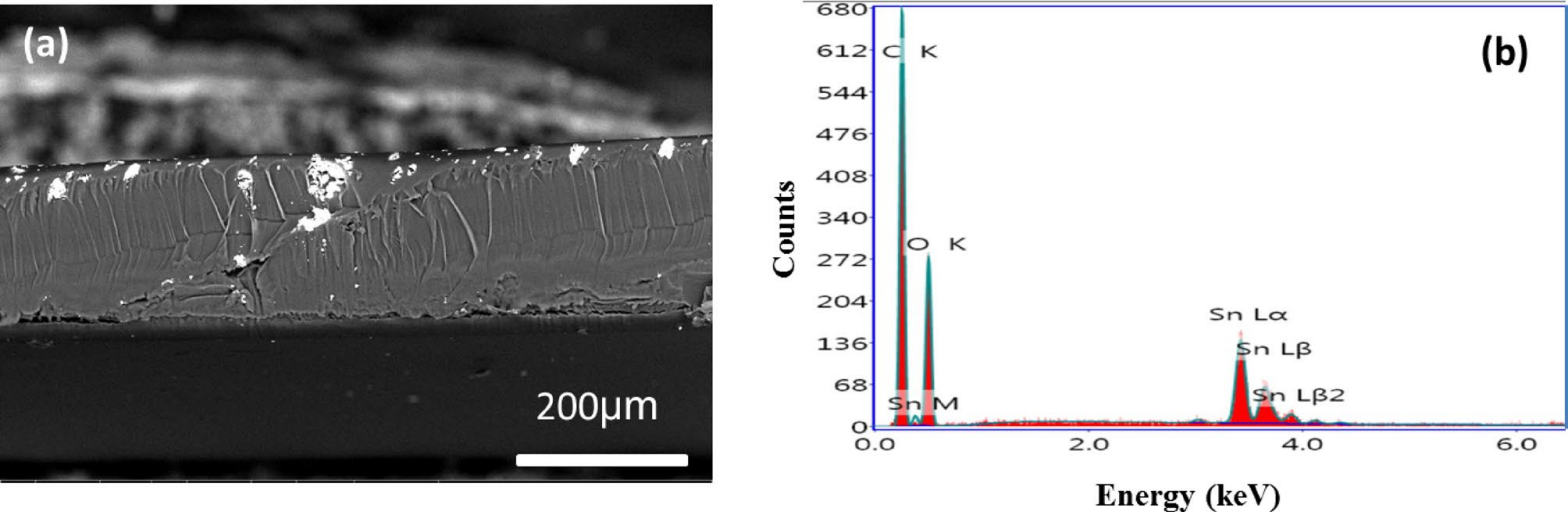
**(a)** SEM cross-sectional image of PVA/SnO − 5% film and its EDX **(b)**.

The surface morphology of the films is analyzed using SEM to evaluate effects of surface modification. Figure 4 (a-c) presents the SEM images of pristine PVA, PVA/SnO − 2% and PVA/SnO − 5% films, respectively. Figure 4a reveals a smooth and homogeneous surface of the pure PVA film, with no observable particles. In contrast, Fig. 4b demonstrates the incorporation of smaller SnO particles at lower concentrations (2 wt %), which exhibit minimal impact on the overall morphology of the composite film. However, as the SnO concentration increases to 5 wt%, distinct particle aggregation and the formation of distribution clusters are observed on the PVA surface, as shown in Fig. 4c. This aggregation is accompanied by a significant increase in particle size and a reduction in the mean interparticle distance. According to Goud et al., this phenomenon can be attributed to the agglomeration of PVA chain molecules, leading to the formation of large, intensely white-colored particles. The enhanced surface area of SnO nanoparticles, due to their nano-filler properties, makes them suitable for applications such as gas sensing in detection devices [40]. Furthermore, Fig. 5a provides a cross-sectional SEM image of the PVA/SnO − 5% film, highlighting the uniform dispersion of SnO particles within the polymer matrix. The samples thicknesses are determined through direct measurement of cross-sectional SEM image. Using the image analysis software (Image J), thickness values are obtained at five spatially distributed locations across each nanocomposite film. The reported thickness of 200 ± 5 μm represents the mean values with standard deviation, ensuring statistical reliability for subsequent optical property calculations. EDX analysis of the PVA/SnO − 5% film, depicted in Fig. 5b, confirms the presence of carbon (C), oxygen (O), and tin (Sn) as the sole elemental components, validating the composition of the composite film. Thus, the structural results (XRD, FT-IR, Raman spectra, SEM images, and EDX analysis) confirm the successful incorporation of SnO into PVA matrix.

### Optical characterizations of pva/sno nanocomposite films

#### Optical band gap

**Fig. 6:**
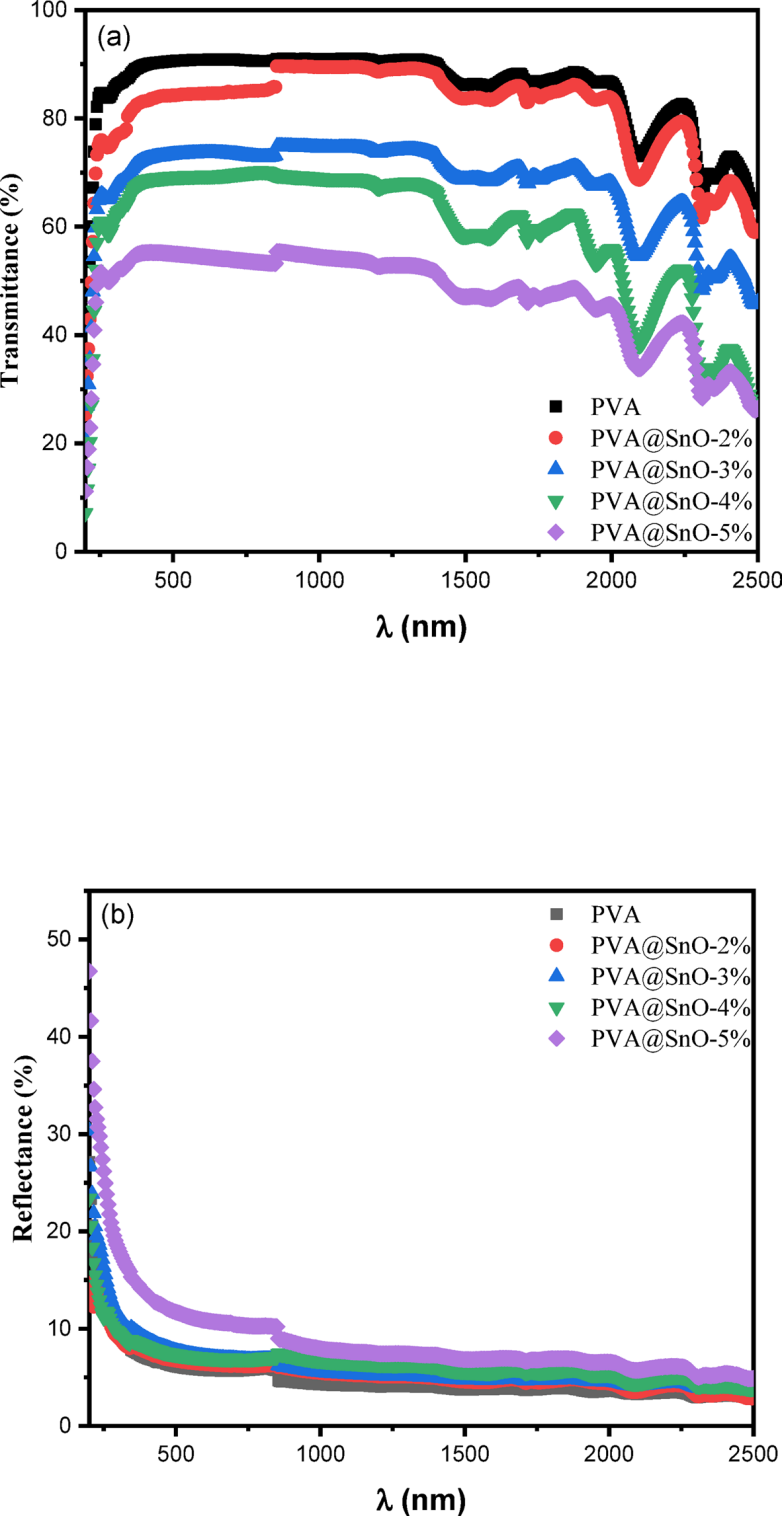
Plots for the PVA and PVA films doped with SnO: **(a)** transmittance vs. wavelength and **(b)** reflectance versus wavelength.

The transmittance, T (λ), and reflectance, R (λ), for pure PVA and PVA/SnO nanocomposite films are investigated over the wavelength range of 200–2500 nm. The results, as depicted in Fig. 6(a), indicate that the concentration of SnO significantly influences the reduction in transmittance and induces a shift in the absorption edge (cut-off wavelengths). It is generally observed that film transmittance decreases with increasing grain size in the visible region of the spectrum, primarily due to light scattering on rough surfaces. The pure PVA film exhibits high transparency, with a transmittance of approximately 91.14% at a wavelength of 400 nm. However, the transmittance decreases progressively to 84.77, 74.2, 69.8, and 56.55% for composite films containing 2, 3, 4, and 5% SnO, respectively. In the reflectance spectra (Fig. 6(b)), a sharp decrease in reflectance is observed in the 200–250 nm regions, which is associated with the material’s band gap and suggests the semicrystalline nature of PVA^46^. As the SnO concentration increases, the reflectance also increases. Additionally, visual observation reveals a gradual change in the films color from transparent for pure PVA to semitransparent with the incorporation of SnO. This color change indicates that the nanocomposite materials absorb light across a broad spectral range, further confirming the influence of SnO on the optical properties of the PVA matrix. The T (λ) can be used to evaluate the absorption coefficient (α) in accordance with the following equation^47^:

**Fig. 7:**
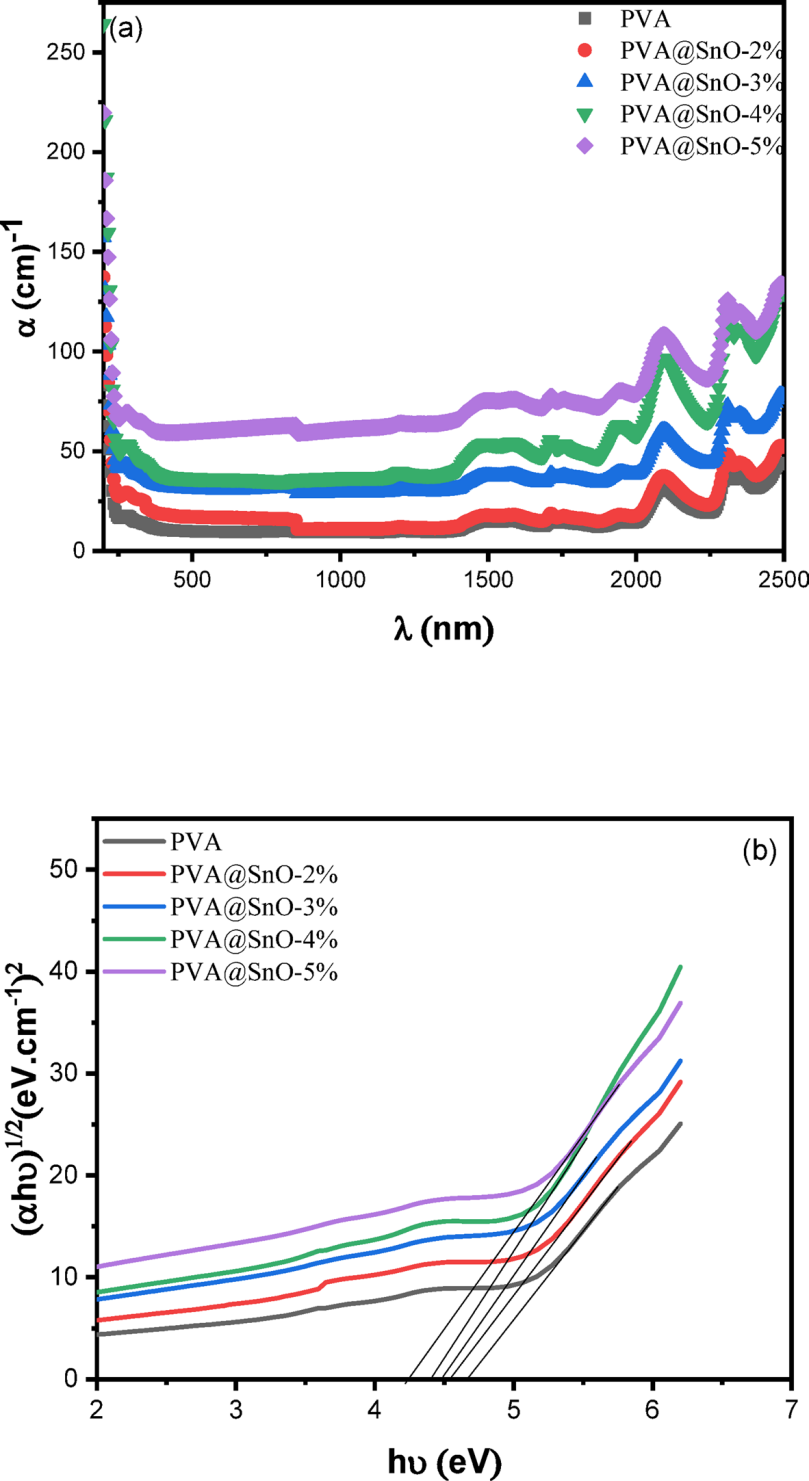
Absorbance coefficient (*α*) vs. wavelength and (*αhν*) ^1/2^ vs. *hν* of nanocomposite PVA/SnO film.

6 $$\:{\upalpha\:}=\raisebox{1ex}{$1$}\!\left/\:\!\raisebox{-1ex}{$d$}\right.\text{ln}\left(\raisebox{1ex}{$1$}\!\left/\:\!\raisebox{-1ex}{$T$}\right.\right)$$

where, **d** is the sample thickness. The α vs. wavelength (λ) plots for PVA/SnO films at various content of SnO are shown in Fig. (7a). The absorption spectra close to the absorption edge were used to calculate the optical energy band gap of the films. The expression for the absorption coefficient’s dependency on photon energy is^47,48^:

7 $$\:{\upalpha\:}\text{h}{\upnu\:}=\text{A}{\left(\text{h}{\upnu\:}-{\text{E}}_{\text{g}}^{\text{O}\text{p}\text{t}}\right)}^{\text{r}}$$

where, $\:{E}_{g}^{Opt}$ is optical energy band gap, r is an exponent that have values of 1, 2, 3, 1/2, or 3/2 depending on the type of electronic transitions causing the optical absorption, A is a constant, and hυ is the energy of the photon. Values of optical energy gap for each electronic transition are obtained by extrapolating the straight lines parts of curves to $\:{\left(\alpha\:h\nu\:\right)}^{1/2}=$0 (see Fig. (7b)) and are tabulated in Table 2. It is clearly shown that when the SnO concentration increases, the optical band gap values decrease. The obtained results demonstrate that the bandgap structure of SnO/PVA nanocomposites can be effectively modulated by varying the SnO-to-PVA ratio within the blend. Furthermore, the incorporation of SnO facilitates the formation of a three-dimensional (3D) networked structure within the polymer matrix, creating conductive pathways that enhance the semiconducting properties of the composite^49^. This structural modification leads to a reduction in the optical bandgap from 4.59 to 4.18 eV, as evidenced by the data presented in Table 2. These findings highlight the potential of SnO as functional filler for tailoring the electronic and optical properties of PVA-based materials. The comparison of $\:{E}_{g}^{Opt}\:$values shown in Table 3 provides important information about our study compared to earlier research. In this study on PVA/SnO nanocomposites, it is different from earlier research because it uses a small amount of SnO nano-fillers (2–5 wt%), which results in a significant drop in the indirect $\:{E}_{g}^{Opt\:}\:$to 4.18 eV. This improvement contrasts with reported nanocomposites employing diverse fillers (e.g., Al₂O₃, CaThO₃, Co⁺, CuO, Fe₂O₃, NiO, TiO₂)^43,50–53^which often required higher filler loadings (e.g., up to 10 wt% for Co⁺) to attain comparable bandgap modulation. The effectiveness of SnO at this lower amount leads to $\:{E}_{g}^{Opt}$ values that are as good as those from well-known fillers like TiO₂, Al₂O₃^50^and CuO^43^ NPs, while also providing important practical benefits: using less filler lowers production costs and greatly reduces the chance of harmful clumping of the nano-fillers that can happen with higher amounts. This work shows that SnO is useful filler for adjusting the bandgap in PVA-based nanocomposites, enhancing their optical performance and making the materials cheaper and more reliable.

**Table 2:** The dispersion, dielectric and nonlinear parameters pva/sno films.

Estimated parameters	PVA film	PVA − 2% SnO film	PVA − 3% SnO film	PVA − 4% SnO film	PVA- 5% SnO film
**(** $\:{\mathbf{E}}_{\mathbf{g}}^{\mathbf{O}\mathbf{p}\mathbf{t}}$ **) eV**	4.59	4.47	4.3	4.25	4.18
**(E**_**o**_ **) eV**	6.28	6.05	5.86	5.75	5.53
**(E**_**d**_ **) eV**	9.28	10.21	11.25	12.25	14.17
$\:{\varvec{\epsilon\:}}_{\mathbf{\infty\:}}^{\varvec{W}\varvec{D}}$	2.47	2.68	2.91	3.13	3.56
$\:{\varvec{n}}_{\varvec{o}}$	1.57	1.63	1.71	1.76	1.88
**So (m**^**−2**^ **) x 10**^**13**^	0.37	0.45	3.8	5.07	5.53
**λ**_**o**_ **(nm)**	391	358	45.16	49.76	32.25
$\:{\varvec{\epsilon\:}}_{\varvec{L}}$	2.35	2.66	2.69	2.98	3.28
$\:\frac{\varvec{N}}{{\varvec{m}}^{\mathbf{*}}}\:$ **x10** ^**49**^	6.599	12.298	13.63	14.302	17.31
$\:{\varvec{n}}_{2}\:\:\:$ **x 10** ^**−12**^ **(hυ → 0)**	0.779	1.27	2.04	2.99	5.87
$\:{\varvec{\chi\:}}^{\left(3\right)}\:$ **x 10** ^**−13**^ **(hυ → 0)**	0.325	0.554	0.928	1.41	2.94

**Table 3:** Comparative analysis of the variation in indirect optical energy gap values obtained in this study and those reported in the literature^43,50–53^ for the PVA polymer blend filled with various fillers.

Polymers matrix	Filler	Filler’s ratio (wt %)	Eg (eV)	Refs.	
PVA	CuO	0-0.4	5.35–4.5	^43^	
PVA	Al_2_O_3_	1–3	4.94–4.89	^50^	
PVA	CaThO_3_	0–4	5.08–4.62	^51^	
PVA	Co+	0.1–10	4.96–4.01	^52^	
PVA	Fe_2_O_3_	1	4.8	^53^	
PVA	NiO	1	5	^53^	
PVA	SnO	2–5	4.59–4.18	Present work	

#### Photoluminescence properties for pva/sno film

**Fig. 8:**
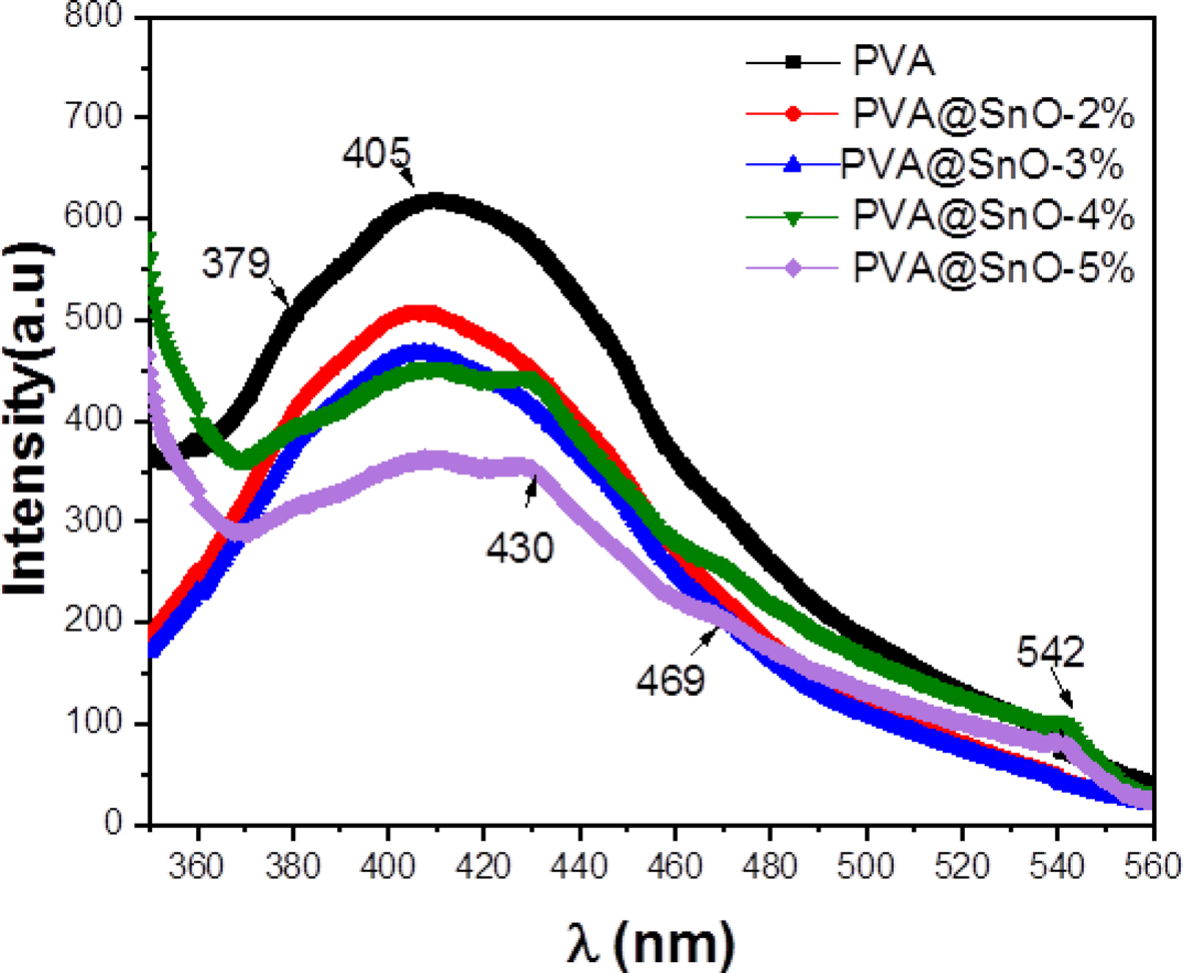
PL vs. wavelength (nm) for nanocomposite PVA/SnO film.

The photoluminescence (PL) emission spectra of pure PVA and PVA/SnO (2, 3, 4 and 5%) are examined, as showed in Fig. 8. The PL spectra are gotten using an excitation wavelength of 330 nm. The PVA film showed a separate luminescence peak within the visible spectrum, ranging from 350 to 550 nm. The peak at 379 nm is attributed to the electronic transition (π* → n) of the hydroxyl groups in PVA, though the peak at 405 nm is linked with (π → π*) electronic transitions of the free hydroxyl (–OH) groups inside the PVA matrix^54,55^. The introduction of SnO into the PVA matrix resulted in a significant quenching of the luminescence intensity. The formation of cross-linking interactions between the (–OH) groups of PVA and the oxygen atoms of SnO is responsible for this quenching result^54,55^. The interactions change the electronic environment of the PVA matrix, which reduces the processes that create light, known as radiative recombination. The amount of quenching is found to be concentration-dependent, with higher SnO concentrations inducing more pronounced quenching effects. Specifically at (4 and 5%) films showed three emission peaks centered at approximately 432, 469, and 542 nm. The emission band at 432 nm is associated with the band emission of SnO, consistent with its band gap range of 2.7–3.4 eV^26,27^. The peaks at 469 and 542 nm are likely related to defects such as oxygen vacancies. As a result, the PL spectrum of PVA/SnO seems to include influences from both PVA and SnO nanoparticles^29,56,57^.

**Fig. 9:**
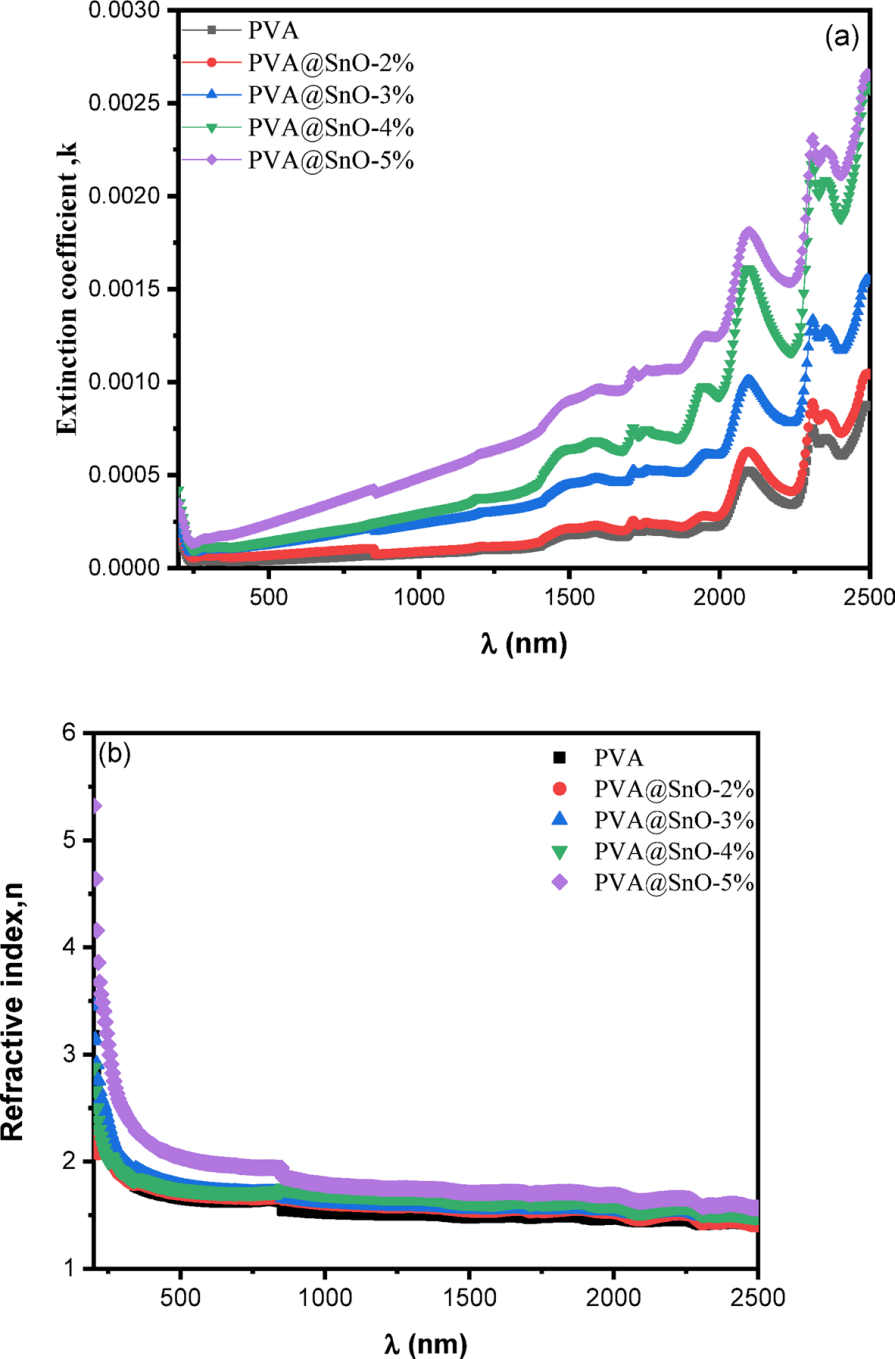
Extinction coefficient, k **(a)**, and Reflective index n **(b)** for PVA/SnO films.

**Fig. 10:**
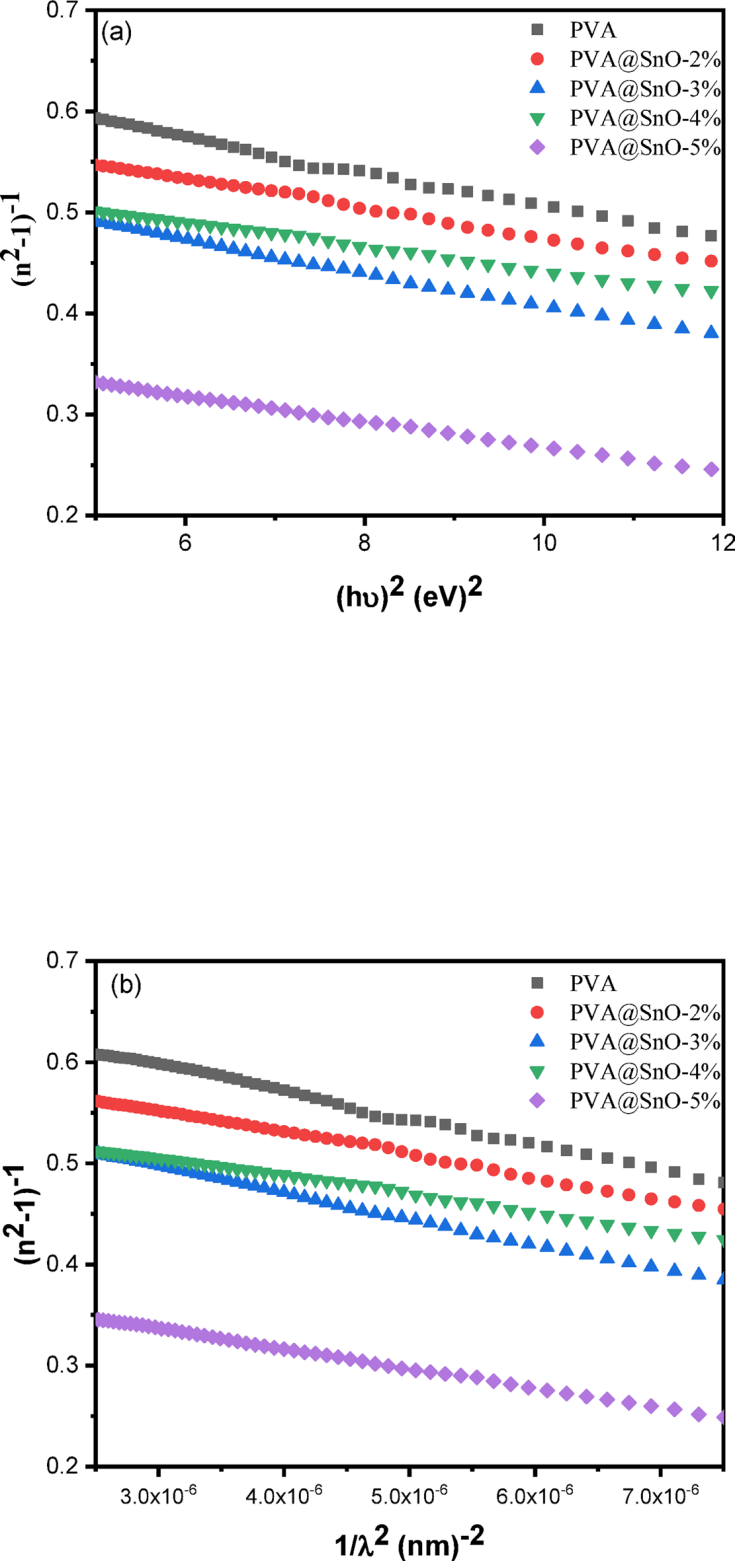
**(a)** Plots of (n^2^−1) ^−1^ vs. (hν)^2^**(b)** Plots of (n ^2^−1) ^−1^ vs. λ^−2^ for PVA-SnO films.

**Fig. 11:**
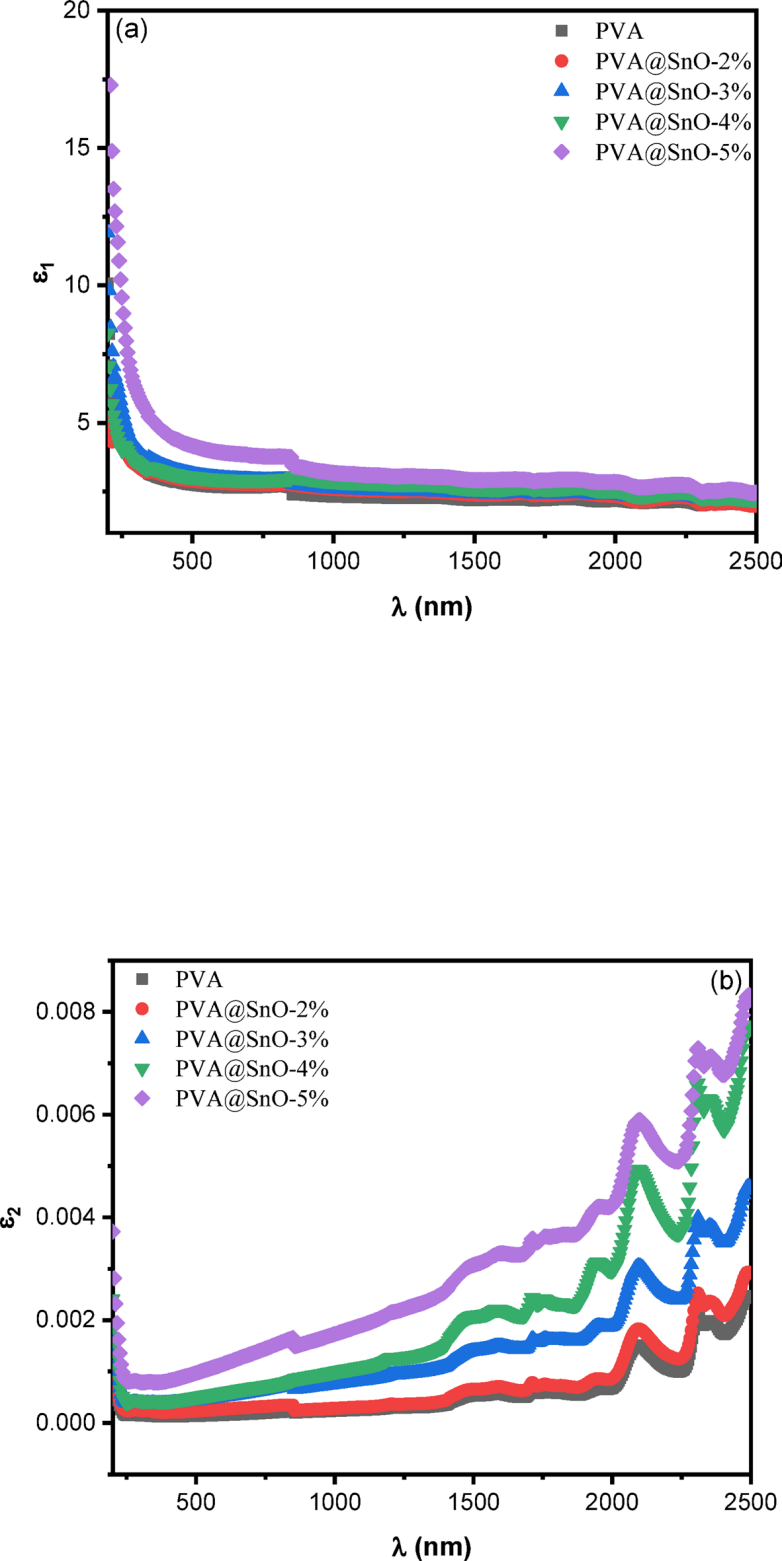
The variation of **(a)** real (ε_1_) and **(b)**, imaginary (ε_2_) parts of the dielectric constant vs. the wavelength for PVA/SnO films.

**Fig. 12:**
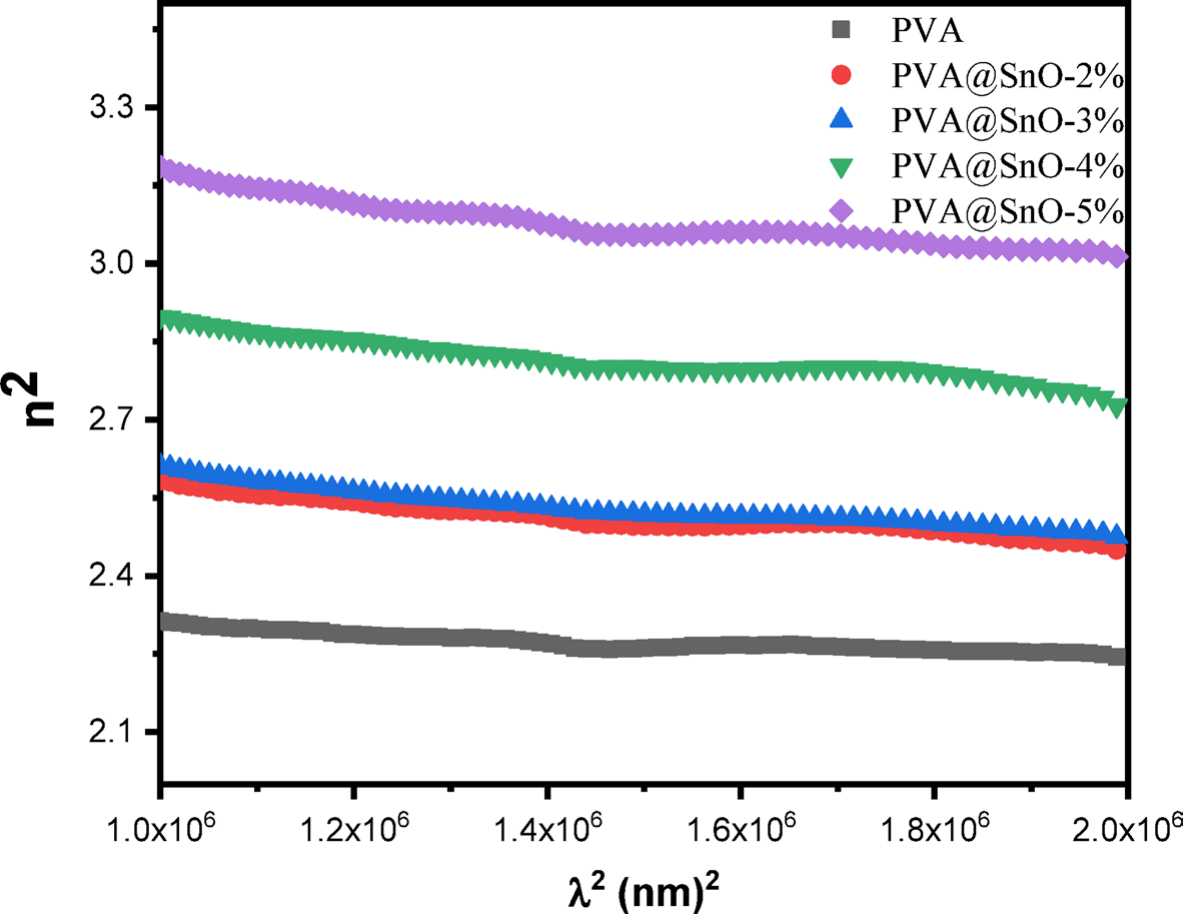
Plots of n^2^ vs. λ^2^ of the for PVA/SnO films.

#### Dispersion and optoelectrical parameters

The absorption coefficient (α) and reflectance R (λ) are used to compute optical constants refractive index n and the extinction coefficient, k, using the following formulae^58,59^ :

8 $$\:\begin{array}{c}k=\frac{{\upalpha\:}\:\lambda\:}{4\pi\:}\\\:\:\end{array}\:\:$$

9 $$\:n=\frac{1+R}{1-R}+\sqrt{\frac{4R}{{\left(1-R\right)}^{2}}-{k}^{2}}$$

Figure (9a) shows the variation in the extinction coefficient k with wavelength for PVA-SnO films with varying SnO content. Extinction coefficient values are lower for PVA films and greater for PVA-SnO films. It was also shown that the deposited nanocomposites films had good optical transparency by the observed low value of extinction coefficient in the visible-NIR wavelength ranges, which showed negligible light loss from scattering^60^. The variation of the refractive index, n, with wavelength for samples is shown in Fig. (9b). It is also observed that an increase in the SnO content leads to a corresponding rise in the refractive index of PVA/SnO composites.

The incorporation of SnO NPs (5 wt%) into the PVA matrix elevates the refractive index from 1.71 (pristine PVA) to 2.16 at λ = 400 nm. This 26% enhancement arises from two synergistic factors: (i) the inherent high polarizability of Sn²⁺ ions and (ii) matrix densification due to SnO/PVA hydrogen bonding (confirmed via FT-IR/Raman). Critically, this performance exceeds that of PVA/SiO₂^61^ nanocomposites (Δn = 18% at 10 wt%) and approaches PVA-CoO-SiO₂^62^ ternary systems (*n* ≈ 2.7 at 6 wt%), despite requiring lower filler content and simpler binary composition. The high refractive index (*n* > 2.0 across visible wavelengths) facilitates enhanced optical confinement, positioning these films as competitive candidates for waveguides, high-efficiency optical filters, and nonlinear photonic devices leveraging intense field-matter interactions^60^.

The high-frequency refractive index of an optical material can be determined using an approximation relation derived from the single oscillator model proposed by Wemple and DiDomenico^63^:

10 $$\:{\left({n}^{2}\left(\text{h}{\upnu\:}\right)-1\right)}^{-1}=\frac{{E}_{o}}{{E}_{d}}-\frac{{\left(h\nu\:\right)}^{2}}{{E}_{o}{E}_{d}}$$

where, E_d_ is the dispersive energy, which represents the average intensity of inter-band optical transitions, and E_o_ is the single oscillator energy. As the (n^2^ − 1) ^−1^ vs. (hv)^2^ plot for PVA-SnO films is shown in Fig. (10a) to be linear, the E_o_ and E_d_ values were calculated using the slopes and intercepts values of these plots, and the results are tabulated in Table (2). When the SnO content is increased from 2 to 5%, it was discovered that the E_d_ values of these films increase from 9.28 to 14.17 eV. While, the E_o_ values declined from 6.28 to 5.53 eV, suggesting a reduction in the average energy gap associated with the oscillator.

SnO/PVA films (5 wt%) exhibit superior dispersion properties and optical performance, evidenced by their significantly higher dispersion energy (E_d_ = 14.17 eV) and single oscillator energy (E_o_ = 5.53 eV) compared to PVA/CS/CuO (5 wt%)^64^ (E_d_ = 2.54 eV, E_o_ = 4.24 eV), PVA/TiO₂^65^(5 wt%) (E_d_ = 11.8 eV, E_o_ = 2.75 eV) and PVA/TiO₂/MWCNT (0.01mg)^66^ (E_d_= 7.75 eV, E_o_ = 2.52 eV). This enhanced E_d_ reflects stronger inter-band optical transitions and charge carrier interactions, while the elevated E_o_ indicates a more ordered electronic structure with reduced defects. Crucially, SnO achieves this with only 5 wt% loading lower than typical filler concentrations in competing systems enabling dramatic reductions in optical bandgap (28% decrease), and minimal nanoparticle aggregation.

This behavior reflects the influence of SnO incorporation on the electronic structure and optical properties of the PVA matrix, highlighting the potential for tailoring these composites for specific optoelectronic. By extending hv → ∞, the Wemple-Didomenico model produces the high-frequency dielectric constant ε_∞_^67,68^:

11 $$\:{\epsilon\:}_{\infty\:}=\sqrt{\left(1+\raisebox{1ex}{${E}_{d}$}\!\left/\:\!\raisebox{-1ex}{${E}_{o}$}\right.\right)}$$

In contrast, the formula for calculating the value of static refractive indices (n_o_) is^68^ $\:{\text{n}}_{\text{o}}=\sqrt{{\epsilon\:}_{\infty\:}}$. The value of n_o_ and ε_∞_ or the PVA/SnO nanocomposite films exhibit an increasing trend with higher SnO content. This behavior is attributed to the enhanced polarizability and densification of the composite structure due to the incorporation of SnO nanoparticles. Such concentration-dependent optical properties make these nanocomposite films highly attractive for photonic applications, including waveguides, and optical filters. The single-term Sellmeier oscillator model may also be used to study the refractive index^68,69^:

12 $$\:{({n}^{2}-1)}^{-1}=\frac{1}{{S}_{o}{\lambda\:}_{o}^{2}}(1-{\lambda\:}_{o}^{2}\:{\lambda\:}^{-2})$$

where, λ_o_ is the average oscillator wavelength and S_o_ is the average oscillator wavelength. Plotting of (n^2^ − 1) ^−1^ as a function of λ^−2^ for the PVA/SnO nanocomposites films is shown in Fig. (10b). Table (2) lists the values of S_o_ and λ_o_, which were calculated from the plots’ linear fitting.

#### Optical dielectric properties for pva/sno nanocomposite films

While a spectrophotometer cannot directly measure dielectric parameters experimentally, these parameters can be calculated using the following equations derived from the **n** and **k** data^70^:

13 $$\:\left\{\begin{array}{c}\epsilon\:={\epsilon\:}_{1}+j{\epsilon\:}_{2}\\\:{\epsilon\:}_{1}={n}^{2}-{k}^{2}\\\:{\epsilon\:}_{2}=2nk\end{array}\right\}$$

where, ε_1​_ and ε_2 ​_represent the real part (optical dielectric constant) and the imaginary part (optical dielectric loss) of the dielectric constant, respectively. Figures (11) illustrates the wavelength dependence of ε_1_ ​and ε_2_​. As shown in Fig. (11a), the ε_1_​ remains nearly constant with increasing wavelength, indicating stable optical properties in the measured range. In contrast, Fig. (11b) reveals that the ε_2_ exhibits a linear increase with wavelength for all samples, reflecting the material’s absorption characteristics. The imaginary part of the dielectric function is closely associated with the absorption coefficient and is primarily influenced by electron transitions from occupied to unoccupied states. Furthermore, as the concentration of SnO nanoparticles in the PVA matrix increases, both ε_1_​ and ε_2_​ show a corresponding rise, as depicted in Fig. (11). This trend aligns with the observed increase in the n and k values. The enhanced dielectric properties with higher SnO content can be attributed to the increased polarizability and densification of the nanocomposite films, which improve their optical and electronic responses. These findings highlight the potential of PVA/SnO nanocomposites for applications in optoelectronic devices, such as sensors, photodetectors, and energy storage systems, where tunable dielectric properties are essential. The ability to control these properties through SnO concentration further underscores the versatility of these materials for advanced technological applications. The relation between the ε_1_ and λ^2^ in the transparent region can be given by^69,71^:

14 $$\:{{\epsilon\:}_{1}=n}^{2}-{k}^{2}={\epsilon\:}_{L}-\left(\frac{{e}^{2}}{4{\pi\:}^{2}{\epsilon\:}_{o}{c}^{2}\:}\right)(N/{m}^{*}){\lambda\:}^{2}$$

where, ε_L_​ is the lattice dielectric constant, e is the elementary charge, c is the speed of light, and N/m* represents the ratio of carrier concentration to the effective mass. Values of ε_L_​and N/m* are derived from the intercept (λ^2^ = 0) and the slope of the linear fit, respectively for PVA/SnO nanocomposite films (see Fig. (12)), these values are summarized in Table 2.

From the Table 2, it is evident that ε_L_​increases with higher SnO content; indicating enhanced dielectric properties due to the incorporation of SnO nanoparticles. Additionally, the values of ε_L_​ are consistently higher than those of the dielectric constant (ε_∞_​) at high-frequency. This discrepancy can be attributed to the increase in free carrier concentration within the nanocomposite films, which contributes to the lattice polarization and overall dielectric response^66^. The rise in free carrier concentration with increasing SnO content further enhances the material’s electrical and optical properties, making it suitable for applications in optoelectronics and photonics. The ability to tune ε_L_ and N/m* by varying the SnO concentration underscores the potential of PVA/SnO nanocomposites for advanced technological applications, such as sensors, energy storage devices, and optical coatings. These findings highlight the critical role of SnO nanoparticles in modifying the dielectric and electronic properties of the PVA matrix, offering a pathway for designing materials with tailored functionalities.

The energy loss characteristics of a material can be described using two key functions: the surface energy loss function (SELF) and the volume energy loss function (VELF). The SELF represents the energy loss experienced by electrons interacting with the material’s surface, while the VELF corresponds to the energy loss of fast electrons traversing the bulk of the material. Both functions are derived from the ε_1_ and ε_2_ are calculated using the following relations^48^:

15 $$\:\text{V}\text{E}\text{L}\text{F}=-\text{l}\text{m}\left(\frac{1}{{\epsilon\:}^{*}}\right)\frac{{{\upepsilon\:}}_{2}}{\left({{\upepsilon\:}}_{1}^{2}+{{\upepsilon\:}}_{2}^{2}\right)}$$

16 $$\:\text{S}\text{E}\text{L}\text{F}=-\text{l}\text{m}\left(\frac{1}{{\epsilon\:}^{*}+1}\right)\frac{{{\upepsilon\:}}_{2}}{\left({\left({{\upepsilon\:}}_{1}^{2}+1\right)}^{2}+{{\upepsilon\:}}_{2}^{2}\right)}$$

**Fig. 13:**
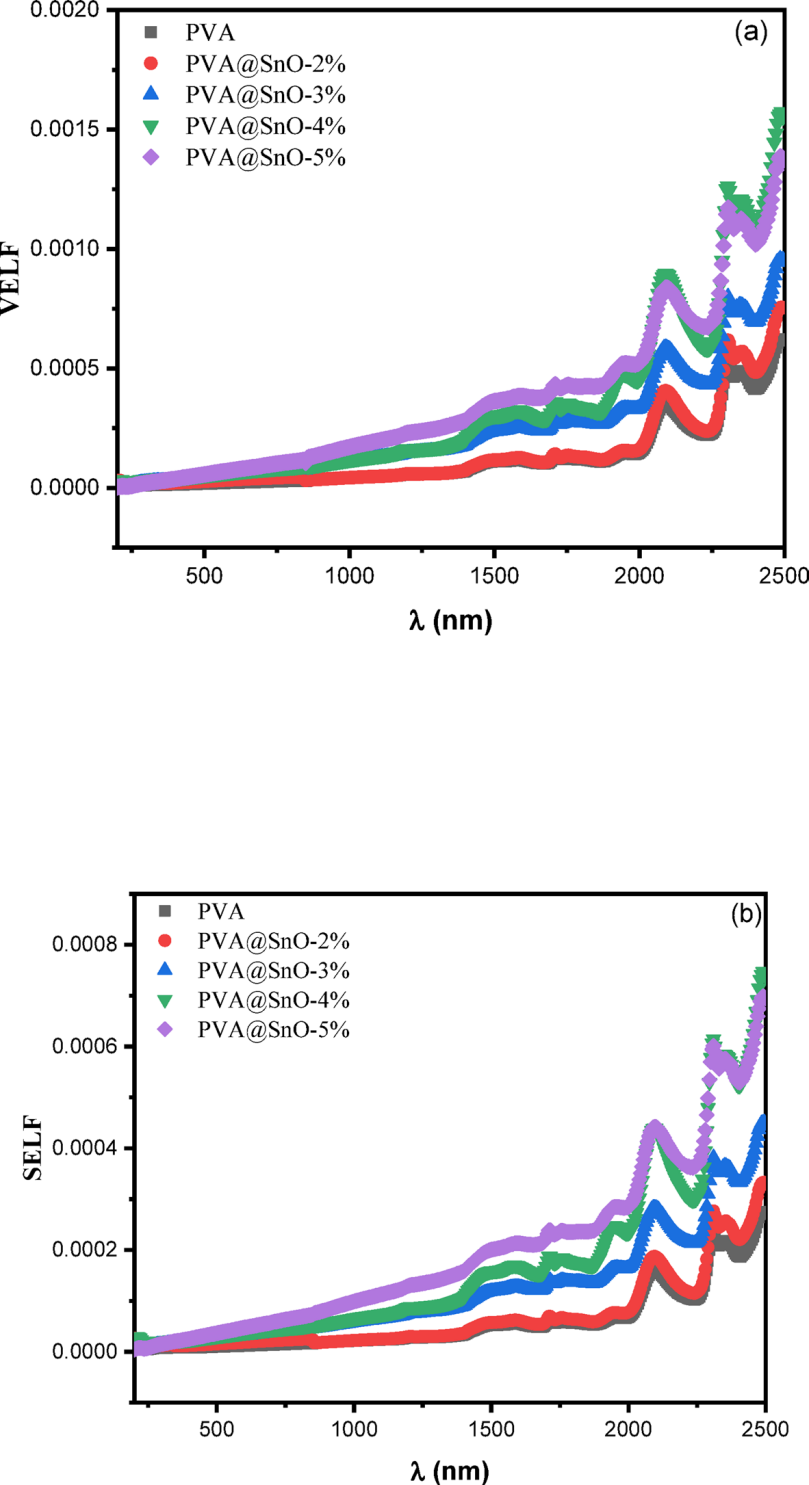
The variation of **(a)** VELF & **(b)** SELF vs. the wavelength for PVA/SnO films.

**Fig. 14:**
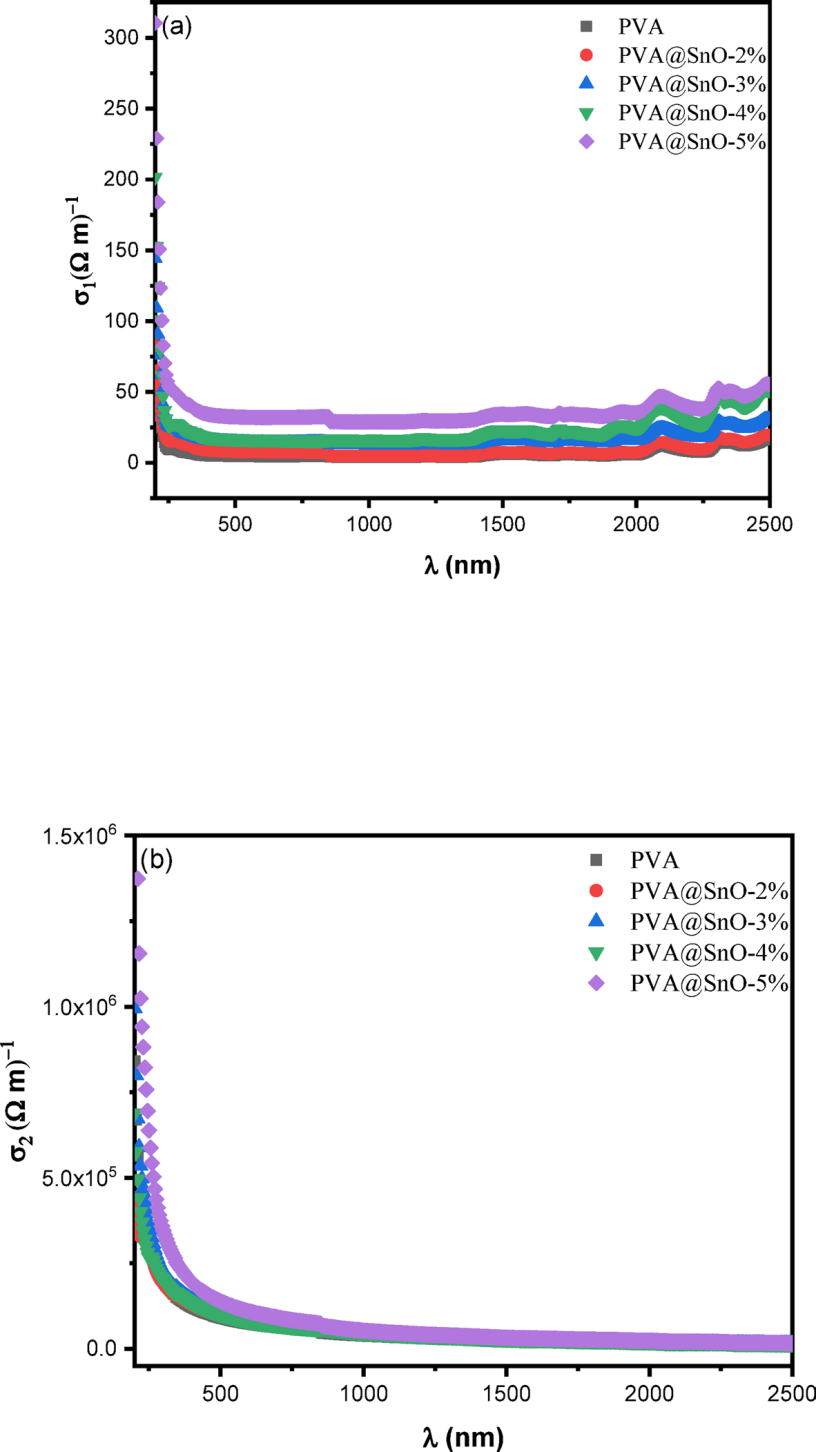
The variation of **(a)** real (σ_1_) and **(b)** imaginary (σ_2_) parts of the optical conductivity vs. the wavelength for PVA/SnO films.

The VELF provides insights into the bulk electronic properties and energy dissipation mechanisms within the material, while the SELF is particularly useful for understanding surface interactions and plasmonic effects. These functions are critical for analyzing the optical and electronic behavior of materials, especially in applications involving electron energy loss spectroscopy (EELS) or surface plasmon resonance (SPR). The dependence of VELF and SELF on ε_1_​ and ε_2_​ highlights the importance of dielectric properties in determining energy loss mechanisms. For instance, materials with high ε_2_​ values typically exhibit stronger energy dissipation due to increased absorption and electronic transitions. By studying these functions, researchers can gain valuable insights into the material’s optical response, electronic structure, and potential applications in fields for example plasmonics, optoelectronics, and energy harvesting. The overall energy loss is shown by the values of VELF and SELF. Figure 13(a&b) shows VELF and SELF verse wavelength for PVA/SnO nanocomposites films. SELF and VELF exhibits similar pattern. Additionally, at the particular peak, VELF increases more than SELF. Furthermore, this figure illustrates how the content of SnO affects the peak locations and intensities of VELF and SELF for PV/SnO nanocomposite films. The optical conductivity (σ) of a material is a fundamental parameter used to characterize its optical response and is calculated using the following relation^72^:

17 $$\:\left\{\begin{array}{c}\sigma\:={{\upsigma\:}}_{1}+j{{\upsigma\:}}_{2}\\\:{{\upsigma\:}}_{1}=\omega\:{{\upepsilon\:}}_{\text{o}}{{\upepsilon\:}}_{2}\\\:{{\upsigma\:}}_{2}=\omega\:{{\upepsilon\:}}_{\text{o}}{{\upepsilon\:}}_{1}\end{array}\right\}$$

where, σ_1_​ and σ_2_​ represent the real and imaginary parts of the optical conductivity, respectively, ω is the angular frequency of the incident light, and ε_o_​ is the permittivity of free space. The optical conductivity is closely related to the dielectric properties of the material, as it depends on both the ε_1_ and ε_2_. Figure 14 (a&b) illustrates the variation in optical conductivity as a function of wavelength for PVA/SnO nanocomposite films. It is observed that the optical conductivity increases with higher SnO content, particularly in the wavelength range below 250 nm. This rise in σ values indicates a highly excited electronic state or enhanced absorbance in the material, suggesting improved charge carrier generation and transport properties^73^. However, as the photon energy increases beyond the absorption edge, the optical conductivity decreases sharply, which corresponds to the optical band gap of the material. This behavior is consistent with the optical band gap values presented in Table 2, confirming the influence of SnO doping on the electronic structure of PVA. The decrease in optical conductivity at higher photon energies can be attributed to the reduced availability of electronic states for excitation, as well as potential structural modifications induced by the charge ordering effect. These structural changes, which are critical in determining the optical properties of the films, may arise from the incorporation of SnO nanoparticles into the PVA matrix. The enhancement in optical conductivity with increasing SnO content highlights the potential of these nanocomposites for optoelectronic applications, such as solar cells, photodetectors, and light-emitting devices, where efficient charge carrier generation and transport are essential. The study of optical conductivity provides valuable insights into the electronic and optical behavior of PVA/SnO nanocomposites, demonstrating their tunable properties and potential for advanced technological applications.

#### Nonlinearity in pva/sno nanocomposites films

Nonlinear optics (NLO) explores phenomena arising from the interaction of materials with high-intensity light, playing a critical role in photonics applications such as optical information processing, data storage, and sensor technologies. Organic materials, particularly those with π-conjugation, often exhibit strong NLO responses due to their electronic structure. In PVA/SnO nanocomposite films, the NLO properties can be analyzed through the linear and nonlinear optical susceptibilities. The linear optical susceptibility (χ ^(1)^) is determined using the next empirical relation based on the linear refractive index (n)^72,73^:

18 $$\:\chi^{(1)}\:=\:(\text{n}^2-1)/4\pi$$

The third-order nonlinear susceptibility (χ^(3)^), which governs effects such as third-harmonic generation (THG), can be determined using a generalized form of Miller’s rule^73,74^:

19 $$\:\:\chi^{(3)} = \text{A}\:(\chi^{(1)})^4$$

where, A = 1.7 × 10^−10^ is a material-independent constant. The nonlinear refractive index ($\:{n}_{2}$) is related to χ ^(3)^ and the static refractive index (n_o_) by the following equation^75^:

20 $$\:{n}_{2}=\frac{12\pi\:{\chi\:}^{\left(3\right)}}{{n}_{o}}$$

**Fig. 15:**
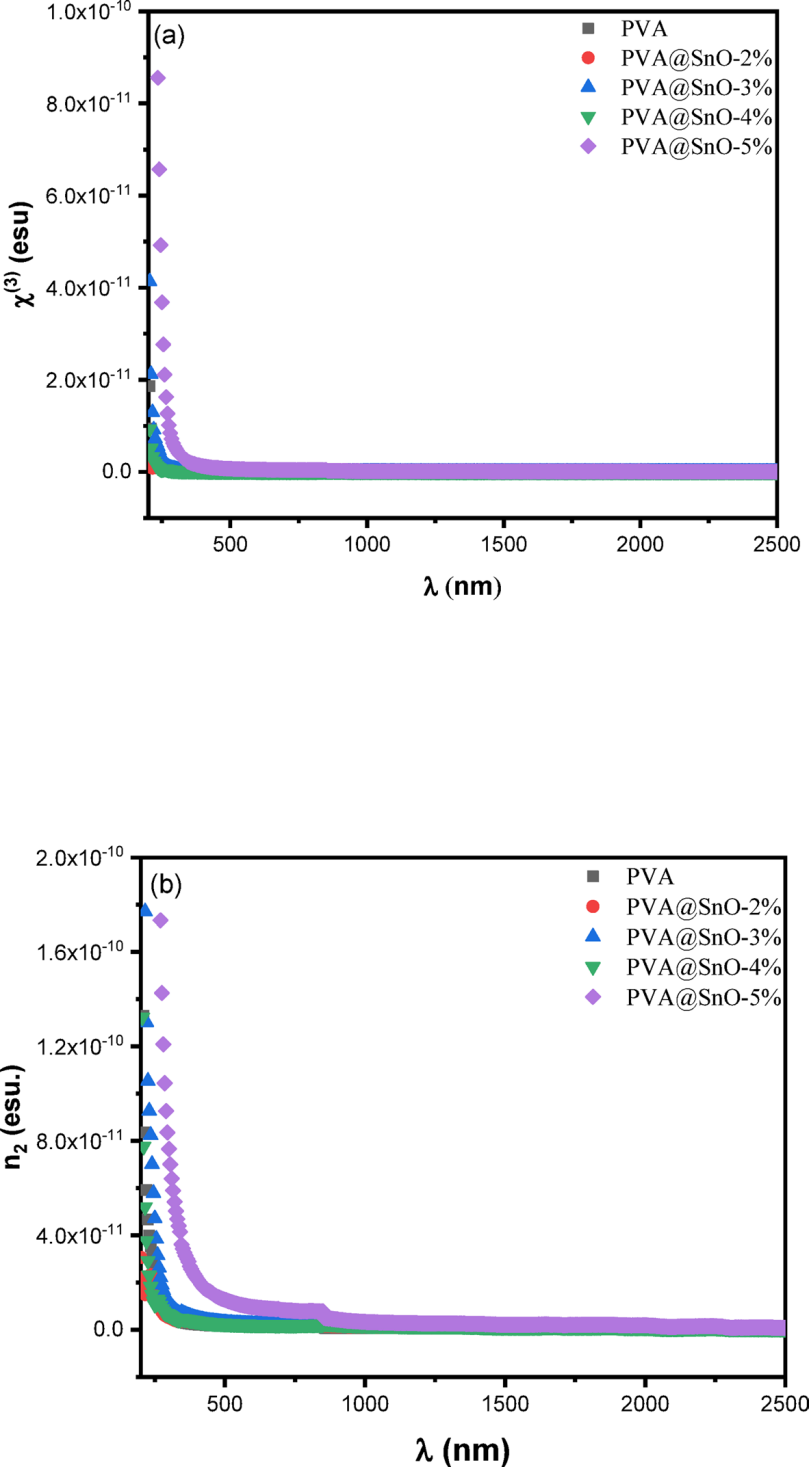
The variation of **(a)** The third order non-linear susceptibility- χ^(3)^ and **(b)** The nonlinear refractive- n_2_ vs. the wavelength for PVA/SnO films.

Figure 15 (a&b) illustrates the variation of χ ^(3)^ and $\:{n}_{2}\:\:$with wavelength for PVA/SnO nanocomposite films. Both χ ^(3)^ and $\:{n}_{2\:}$exhibit a decreasing trend with increasing wavelength. The calculations of $\:{\chi\:}^{\left(3\right)}$ and, $\:{n}_{2}$ at the zero-frequency (hυ = 0) using Eqs. 19&20 are tabled in Table 2. However, as the SnO content increases in the films, Values of χ^3^ and $\:{n}_{2}\:$rise significantly. SnO/PVA films exhibit a significant enhancement in third-order nonlinear optical response relative to PVA/TiO₂/MWCNT^66^ nanocomposites films. At 5 wt% SnO loading, SnO/PVA achieves (χ⁽³⁾) and (n₂) values of 2.94 × 10⁻¹³ esu and 5.87 × 10⁻¹² esu (at hν→0), respectively. This represents enhancement over PVA/TiO₂@MWCNT (χ⁽³⁾ = 20.37 × 10⁻¹⁵ esu, n₂ = 38.42 × 10⁻¹⁴ esu at 0.01 MWCNT). This enhancement in nonlinear optical properties is attributed to the increased polarization of the polymer matrix at higher SnO concentrations, which allows for greater absorption of electromagnetic waves and improved NLO responses^74^. The reduction in the optical band gap with increasing SnO content further contributes to the enhanced nonlinearity, as it facilitates electronic transitions and charge carrier generation. The improved NLO properties of PVA/SnO nanocomposites make them promising candidates for nonlinear optoelectronic devices, such as optical limiters, modulators, and switches. The incorporation of SnO nanoparticles into the PVA matrix introduces Sn²⁺ free ions with high polarizability and bonded oxygen atoms, which are responsible for the observed increase in nonlinear optical parameters^76^. These findings suggest that increasing the SnO content in the polymer matrix is an effective strategy for tailoring the NLO properties of the material, making it suitable for advanced photonic and optoelectronic applications. The tunable nonlinear optical properties of PVA/SnO nanocomposite films, driven by SnO concentration, highlight their potential for use in next-generation optical technologies. The interplay between structural modifications, electronic transitions, and polarizability underscores the importance of these materials in the field of nonlinear optics.

## Conclusions

In this work, we systematically studied structural, optical, and photoluminescence properties of PVA/SnO nanocomposite films, synthesized via a solution-casting technique. XRD confirmed that SnO incorporation reduced PVA’s amorphous phase, increasing crystallite size by 28% (25.74 → 32.88 nm) while lowering dislocation density (1.51 × 10¹⁵ → 0.92 × 10¹⁵ m⁻²). Homogeneous NP dispersion (< 3 wt %) transitions to aggregation at 5 wt% (SEM), establishing a critical loading threshold. FTIR spectroscopy confirmed the successful integration of SnO nanoparticles into the PVA matrix, with distinct vibrational peaks corresponding to Sn-O bonds observed at 598 cm^−1^. In addition, Specific E_g_ and A_₁g_ modes of SnO at 109 and 208 cm^⁻¹^ were confirmed by Raman spectra. The band gap narrows from 4.59 eV (pristine PVA) to 4.18 eV (5 wt% SnO), enhancing semiconductivity. PL spectra revealed concentration-dependent quenching via PVA–SnO cross-linking, with new defect-mediated emissions at 469/542 nm. Furthermore, the optical dielectric properties showed a rise with the higher SnO content, indicating enhanced polarizability and densification of the nanocomposite films. The nonlinear optical properties, such as χ ^(3)^ and n_2_, also exhibited a significant enhancement with increasing SnO concentration, making these materials promising candidates for use in flexible optoelectronic and nonlinear optical devices.

## Data Availability

All data generated or analyzed during this study are included in the manuscript. Additional data or data files can be provided by the corresponding author upon request.
